# 2-Propyl 3,3-dibromo-2-hydroxy­pyrrolidine-1-carboxyl­ate

**DOI:** 10.1107/S1600536810005106

**Published:** 2010-02-13

**Authors:** Gary S. Nichol, Steven Gunawan, Zhigang Xu, Justin Dietrich, Christopher Hulme

**Affiliations:** a Department of Chemistry and Biochemistry, The University of Arizona, 1306 E. University Boulevard, Tucson, AZ 85721, USA; b Southwest Center for Drug Discovery and Development, College of Pharmacy, BIO5 Institute, University of Arizona, Tucson, AZ 85721, USA

## Abstract

The title compound, C_8_H_13_Br_2_NO_3_, crystallizes as a non-merohedral twin with twin law −0.6 0 0.4/0 − 1 0 /1.6 0 0.6, and the structure has a refined twin domain ratio of 0.546 (5). The structure shows a compact conformation, with the ester unit roughly coplanar with a mean plane fitted through the non-H atoms of the pyrrolidine ring [dihedral angle = 8.23 (9)°]. In the crystal, inversion dimers linked by pairs of O—H⋯O hydrogen bonds generate an *R* ^2^_2_(12) motif.

## Related literature

For details of the synthesis, see: Magnus *et al.* (1994 ▶); Salamant & Hulme (2006 ▶). For puckering parameters, see: Cremer & Pople (1975 ▶). For hydrogen-bonding motifs, see: Bernstein *et al.* (1995 ▶).
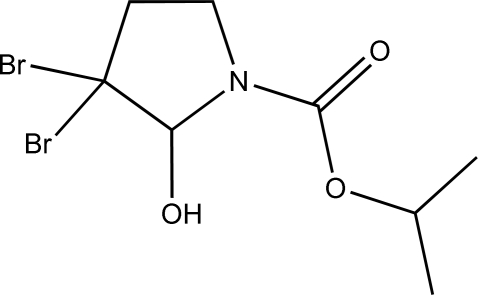


## Experimental

### 

#### Crystal data

C_8_H_13_Br_2_NO_3_
*M* *_r_* = 331.01
Monoclinic, 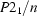

*a* = 10.1061 (5) Å
*b* = 5.9914 (3) Å
*c* = 18.5496 (9) Å
β = 95.880 (2)°
*V* = 1117.26 (10) Å^3^
*Z* = 4
Mo *K*α radiation
μ = 7.24 mm^−1^
*T* = 100 K
0.44 × 0.16 × 0.11 mm

#### Data collection

Bruker Kappa APEXII DUO CCD diffractometer
Absorption correction: multi-scan (TWINABS; Sheldrick, 1996 ▶) *T*_min_ = 0.144, *T*_max_ = 0.514
34994 measured reflections
9572 independent reflections
7956 reflections with *I* > 2σ(*I*)
*R*_int_ = 0.042

#### Refinement

*R*[*F* ^2^ > 2σ(*F* ^2^)] = 0.032
*wR*(*F* ^2^) = 0.082
*S* = 1.03
9572 reflections
138 parameters
H atoms treated by a mixture of independent and constrained refinement
Δρ_max_ = 1.41 e Å^−3^
Δρ_min_ = −0.77 e Å^−3^

### 

Data collection: *APEX2* (Bruker, 2007 ▶); cell refinement: *SAINT* (Bruker, 2007 ▶); data reduction: *SAINT* and *CELL_NOW* (Sheldrick, 2004 ▶); program(s) used to solve structure: *SHELXTL* (Sheldrick, 2008 ▶); program(s) used to refine structure: *SHELXTL*; molecular graphics: *ORTEP-3 for Windows* (Farrugia, 1997 ▶) and *Mercury* (Macrae *et al.*, 2008 ▶); software used to prepare material for publication: *SHELXTL* and local programs.

## Supplementary Material

Crystal structure: contains datablocks I, global. DOI: 10.1107/S1600536810005106/fj2278sup1.cif

Structure factors: contains datablocks I. DOI: 10.1107/S1600536810005106/fj2278Isup2.hkl

Additional supplementary materials:  crystallographic information; 3D view; checkCIF report

## Figures and Tables

**Table 1:** Hydrogen-bond geometry (Å, °)

*D*—H⋯*A*	*D*—H	H⋯*A*	*D*⋯*A*	*D*—H⋯*A*
O1—H1*O*⋯O2^i^	0.83 (3)	1.92 (3)	2.7479 (16)	176 (3)

Symmetry code: (i) 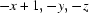.

**Fig. 1.**
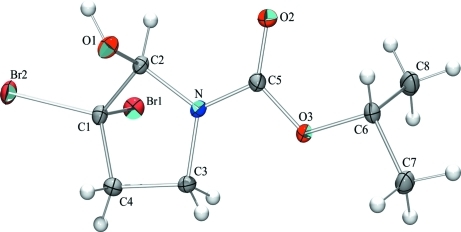
The molecular structure of (I) with anisotropic displacement ellipsoids at the 50% probability level.

**Fig. 2.**
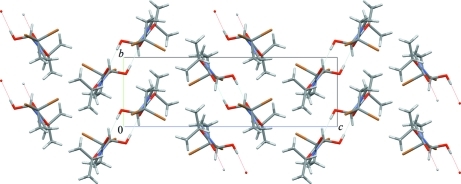
An a-axis packing plot of (I). Blue dotted lines indicate hydrogen bonds; red dotted lines indicate hydrogen bond continuation.

## supplementary crystallographic information

### Comment

We are working to develop new synthetic methodology by application of hypervalent iodine reagents (oxidation state III) in conjunction with bromotrimethylsilane (TMSBr). Specifically, iodosobenzene (Magnus *et al.*, 1994) or iodobenzenediacetate in the presence of TMSBr promotes controlled formation of an oxidized product in one pot, derived from cyclic amides. The transformation represents an α, β, β oxidative process, and the yield of the product, (I), was optimized by varying reaction parameters (Salamant & Hulme, 2006). The structure of this molecule generated from one simple microwave-assisted protocol was confirmed by X-ray crystallography.

The molecular structure of (I) is shown in Fig. 1. Molecular dimensions are unexceptional and the compound has a compact conformation; as evidenced by the torsion angle C2—N—C5—O2 = 0.1 (2)° the ester moiety is essentially co-planar with the pyrrolidine ring. Furthermore the angle between a least-squares plane fitted through all non-hydrogen atoms of the pyrrolidine ring (r.m.s. deviation = 0.1666 Å) and the ester moiety (r.m.s. deviation = 0.0226 Å) is 8.23 (9)°. The pyrrolidine ring adopts an envelope conformation with a Cremer–Pople puckering parameter Q of 0.4053 (15) Å (Cremer & Pople, 1975). The compound forms a hydrogen-bonded dimer by means of an R^2^_2_(12) motif (Fig. 2; Bernstein *et al.*, 1995) although there is no further hydrogen bonding in the crystal structure.

### Experimental

To a solution of isopropyloxypyrrolidine (0.050 g, 0.318 mmol) in anhydrous dichloromethane (1 ml) was added iodobenzene diacetate (0.410 g, 1.272 mmol). Bromotrimethylsilane (0.330 ml, 2.540 mmol) was added dropwise and the mixture irradiated with a Biotage Initiator^TM^ for 20 min at 120°C. The red-brown solution was then dissolved in EtOAc (25 ml) and quenched with 1M Na_2_S_2_O_3_ (2 × 10 ml). The organic layer was washed with 1M NaHCO_3_ (2 × 20 ml), saturated Na_2_CO_3_ (20 ml), brine solution (20 ml), and dried (MgSO_4_). The solvent was evaporated in vacuo and purified by column chromatography (CHCl_3_) to afford the α,β,β product (0.068 g, 0.207 mmol, 65%) as a white solid. MS (+ESI) *m/z* 354 [*M*+Na]^+^

### Refinement

The crystal used was a two-component non-merohedral twin. The two components of the diffraction pattern were easily separated using CELL_NOW (Sheldrick, 2004) with twin law -0.6 0 0.4/0 -1 0 /1.6 0 0.6 and the structure has a refined twin scale factor of 0.546 (5). H atoms were identified from a difference Fourier map. The O—H H atom was freely refined with O—H = 0.83 (3) Å. C—H atoms were refined with U_iso_(H) = 1.5U_eq_(C) (methyl) U_iso_(H) = 1.5U_eq_(C) (all others) with constrained C—H distances in the range 0.98–1 Å.

### Crystal data

**Table:**

C_8_H_13_Br_2_NO_3_	*F*(000) = 648
*M**_r_* = 331.01	*D*_x_ = 1.968 Mg m^−^^3^
Monoclinic, *P*2_1_/*n*	Mo *K*α radiation, λ = 0.71073 Å
Hall symbol: -P 2yn	Cell parameters from 4995 reflections
*a* = 10.1061 (5) Å	θ = 2.2–36.3°
*b* = 5.9914 (3) Å	µ = 7.24 mm^−^^1^
*c* = 18.5496 (9) Å	*T* = 100 K
β = 95.880 (2)°	Rod, colourless
*V* = 1117.26 (10) Å^3^	0.44 × 0.16 × 0.11 mm
*Z* = 4	

### Data collection

**Table:**

Bruker Kappa APEXII DUO CCD diffractometer	9572 independent reflections
Radiation source: fine-focus sealed tube with Miracol optics	7956 reflections with *I* > 2σ(*I*)
graphite	*R*_int_ = 0.042
φ and ω scans	θ_max_ = 36.4°, θ_min_ = 2.2°
Absorption correction: multi-scan (TWINABS; Sheldrick, 1996)	*h* = −16→16
*T*_min_ = 0.144, *T*_max_ = 0.514	*k* = 0→9
34994 measured reflections	*l* = 0→30

### Refinement

**Table:**

Refinement on *F*^2^	Primary atom site location: structure-invariant direct methods
Least-squares matrix: full	Secondary atom site location: difference Fourier map
*R*[*F*^2^ > 2σ(*F*^2^)] = 0.032	Hydrogen site location: difference Fourier map
*wR*(*F*^2^) = 0.082	H atoms treated by a mixture of independent and constrained refinement
*S* = 1.03	*w* = 1/[σ^2^(*F*_o_^2^) + (0.041*P*)^2^ + 0.6397*P*] where *P* = (*F*_o_^2^ + 2*F*_c_^2^)/3
9572 reflections	(Δ/σ)_max_ < 0.001
138 parameters	Δρ_max_ = 1.41 e Å^−^^3^
0 restraints	Δρ_min_ = −0.77 e Å^−^^3^

### Special details

**Table:**

Experimental. 1*H*- NMR (300 MHz, CDCl3) δ ppm 1.26 (d, J = 6.0 Hz, 6H), 2.78 (m, 1H), 3.00 (m, 1H), 3.55 (m, 2H), 4.65 (s, 1H), 5.00 (m, 1H), 5.66 (d, J = 18.6 Hz, 1H)13 C-NMR (75 MHz, CDCl3) δ ppm 22.6, 43.4, 44.6, 64.9, 70.2, 77.9, 155.3
Geometry. All esds (except the esd in the dihedral angle between two l.s. planes) are estimated using the full covariance matrix. The cell esds are taken into account individually in the estimation of esds in distances, angles and torsion angles; correlations between esds in cell parameters are only used when they are defined by crystal symmetry. An approximate (isotropic) treatment of cell esds is used for estimating esds involving l.s. planes.
Refinement. Refinement of *F*^2^ against ALL reflections. The weighted *R*-factor wR and goodness of fit *S* are based on *F*^2^, conventional *R*-factors *R* are based on *F*, with *F* set to zero for negative *F*^2^. The threshold expression of *F*^2^ > σ(*F*^2^) is used only for calculating *R*-factors(gt) etc. and is not relevant to the choice of reflections for refinement. *R*-factors based on *F*^2^ are statistically about twice as large as those based on *F*, and *R*- factors based on ALL data will be even larger.

### Fractional atomic coordinates and isotropic or equivalent isotropic displacement parameters (Å^2^)

**Table:**

	*x*	*y*	*z*	*U*_iso_*/*U*_eq_	
Br1	0.276925 (16)	0.10387 (3)	0.190848 (8)	0.01807 (4)	
Br2	0.113078 (15)	0.17803 (3)	0.039208 (9)	0.01933 (4)	
O1	0.39288 (12)	0.2381 (2)	−0.01016 (6)	0.01668 (19)	
H1O	0.371 (3)	0.120 (5)	−0.0310 (15)	0.029 (7)*	
O2	0.68184 (12)	0.16332 (19)	0.07343 (7)	0.0190 (2)	
O3	0.69480 (10)	0.47225 (18)	0.14461 (6)	0.01432 (18)	
N	0.49624 (12)	0.3442 (2)	0.10291 (7)	0.0134 (2)	
C1	0.27415 (14)	0.2599 (2)	0.09769 (7)	0.0137 (2)	
C2	0.40238 (14)	0.1940 (2)	0.06403 (7)	0.0125 (2)	
H2	0.4265	0.0345	0.0745	0.015*	
C3	0.43486 (14)	0.5339 (2)	0.13709 (8)	0.0153 (2)	
H3A	0.4716	0.6776	0.1219	0.018*	
H3B	0.4479	0.5230	0.1906	0.018*	
C4	0.28818 (15)	0.5100 (2)	0.10886 (8)	0.0156 (2)	
H4A	0.2297	0.5644	0.1448	0.019*	
H4B	0.2673	0.5919	0.0628	0.019*	
C5	0.62874 (14)	0.3155 (2)	0.10427 (8)	0.0137 (2)	
C6	0.84008 (14)	0.4710 (2)	0.14715 (8)	0.0146 (2)	
H6	0.862 (2)	0.423 (4)	0.1000 (12)	0.014 (5)*	
C7	0.88361 (16)	0.7097 (2)	0.16218 (9)	0.0185 (3)	
H7A	0.8399	0.8076	0.1247	0.028*	
H7B	0.9803	0.7206	0.1619	0.028*	
H7C	0.8588	0.7553	0.2097	0.028*	
C8	0.89639 (17)	0.3118 (3)	0.20548 (10)	0.0223 (3)	
H8A	0.9936	0.3090	0.2069	0.033*	
H8B	0.8610	0.1617	0.1949	0.033*	
H8C	0.8711	0.3613	0.2525	0.033*	

### Atomic displacement parameters (Å^2^)

**Table:**

	*U*^11^	*U*^22^	*U*^33^	*U*^12^	*U*^13^	*U*^23^
Br1	0.02072 (7)	0.02068 (7)	0.01338 (6)	−0.00119 (5)	0.00450 (5)	0.00190 (5)
Br2	0.01232 (6)	0.02662 (8)	0.01853 (7)	−0.00274 (5)	−0.00098 (5)	−0.00543 (5)
O1	0.0195 (5)	0.0195 (5)	0.0112 (4)	−0.0029 (4)	0.0021 (4)	−0.0022 (4)
O2	0.0149 (5)	0.0183 (5)	0.0239 (5)	0.0008 (4)	0.0017 (4)	−0.0089 (4)
O3	0.0113 (4)	0.0153 (4)	0.0160 (4)	−0.0003 (3)	−0.0004 (3)	−0.0044 (4)
N	0.0116 (5)	0.0136 (5)	0.0150 (5)	−0.0012 (4)	0.0007 (4)	−0.0046 (4)
C1	0.0126 (5)	0.0162 (5)	0.0119 (5)	−0.0013 (4)	−0.0003 (4)	−0.0009 (4)
C2	0.0120 (5)	0.0142 (5)	0.0113 (5)	−0.0017 (4)	0.0016 (4)	−0.0021 (4)
C3	0.0142 (6)	0.0137 (5)	0.0179 (6)	0.0002 (4)	0.0018 (5)	−0.0047 (5)
C4	0.0148 (6)	0.0145 (6)	0.0173 (6)	0.0018 (4)	0.0010 (5)	−0.0021 (5)
C5	0.0127 (5)	0.0149 (5)	0.0132 (5)	0.0000 (4)	0.0004 (4)	−0.0014 (4)
C6	0.0106 (5)	0.0160 (5)	0.0170 (6)	−0.0002 (4)	0.0009 (4)	−0.0011 (5)
C7	0.0160 (6)	0.0165 (6)	0.0226 (7)	−0.0032 (5)	−0.0006 (5)	0.0003 (5)
C8	0.0187 (7)	0.0180 (6)	0.0286 (8)	0.0002 (5)	−0.0052 (6)	0.0022 (6)

### Geometric parameters (Å, °)

**Table:**

Br1—C1	1.9624 (14)	C3—H3B	0.990
Br2—C1	1.9250 (14)	C3—C4	1.527 (2)
O1—H1O	0.83 (3)	C4—H4A	0.990
O1—C2	1.3949 (17)	C4—H4B	0.990
O2—C5	1.2286 (18)	C6—H6	0.97 (2)
O3—C5	1.3360 (17)	C6—C7	1.513 (2)
O3—C6	1.4641 (18)	C6—C8	1.510 (2)
N—C2	1.4457 (18)	C7—H7A	0.980
N—C3	1.4695 (19)	C7—H7B	0.980
N—C5	1.3478 (19)	C7—H7C	0.980
C1—C2	1.546 (2)	C8—H8A	0.980
C1—C4	1.517 (2)	C8—H8B	0.980
C2—H2	1.000	C8—H8C	0.980
C3—H3A	0.990		
			
H1O—O1—C2	106.6 (19)	C1—C4—H4B	111.3
C5—O3—C6	117.15 (11)	C3—C4—H4A	111.3
C2—N—C3	114.36 (12)	C3—C4—H4B	111.3
C2—N—C5	121.99 (12)	H4A—C4—H4B	109.2
C3—N—C5	123.58 (12)	O2—C5—O3	124.43 (13)
Br1—C1—Br2	108.05 (7)	O2—C5—N	124.50 (13)
Br1—C1—C2	107.27 (9)	O3—C5—N	111.06 (12)
Br1—C1—C4	110.95 (9)	O3—C6—H6	107.0 (13)
Br2—C1—C2	113.74 (9)	O3—C6—C7	105.83 (12)
Br2—C1—C4	112.98 (10)	O3—C6—C8	109.23 (12)
C2—C1—C4	103.71 (12)	H6—C6—C7	111.1 (13)
O1—C2—N	110.45 (12)	H6—C6—C8	110.8 (13)
O1—C2—C1	111.97 (11)	C7—C6—C8	112.58 (13)
O1—C2—H2	111.3	C6—C7—H7A	109.5
N—C2—C1	99.99 (11)	C6—C7—H7B	109.5
N—C2—H2	111.3	C6—C7—H7C	109.5
C1—C2—H2	111.3	H7A—C7—H7B	109.5
N—C3—H3A	111.3	H7A—C7—H7C	109.5
N—C3—H3B	111.3	H7B—C7—H7C	109.5
N—C3—C4	102.54 (11)	C6—C8—H8A	109.5
H3A—C3—H3B	109.2	C6—C8—H8B	109.5
H3A—C3—C4	111.3	C6—C8—H8C	109.5
H3B—C3—C4	111.3	H8A—C8—H8B	109.5
C1—C4—C3	102.32 (11)	H8A—C8—H8C	109.5
C1—C4—H4A	111.3	H8B—C8—H8C	109.5
			
C3—N—C2—O1	99.81 (14)	Br1—C1—C4—C3	73.49 (12)
C3—N—C2—C1	−18.30 (15)	Br2—C1—C4—C3	−165.00 (10)
C5—N—C2—O1	−77.31 (17)	C2—C1—C4—C3	−41.39 (13)
C5—N—C2—C1	164.59 (13)	N—C3—C4—C1	29.33 (14)
Br1—C1—C2—O1	161.81 (9)	C6—O3—C5—O2	5.0 (2)
Br1—C1—C2—N	−81.21 (11)	C6—O3—C5—N	−176.05 (12)
Br2—C1—C2—O1	42.39 (14)	C2—N—C5—O2	0.1 (2)
Br2—C1—C2—N	159.37 (9)	C2—N—C5—O3	−178.85 (12)
C4—C1—C2—O1	−80.72 (13)	C3—N—C5—O2	−176.76 (14)
C4—C1—C2—N	36.26 (13)	C3—N—C5—O3	4.3 (2)
C2—N—C3—C4	−6.68 (16)	C5—O3—C6—C7	152.87 (13)
C5—N—C3—C4	170.38 (13)	C5—O3—C6—C8	−85.69 (15)

### Hydrogen-bond geometry (Å, °)

**Table:**

*D*—H···*A*	*D*—H	H···*A*	*D*···*A*	*D*—H···*A*
O1—H1O···O2^i^	0.83 (3)	1.92 (3)	2.7479 (16)	176 (3)

Symmetry codes: (i) −*x*+1, −*y*, −*z*.
